# Therapeutic Efficacy of Melatonin in Patients with Coronavirus 2019: A Systematic Review and Meta-Analysis of Randomized Controlled Trials: Melatonin in COVID-19 Patients

**DOI:** 10.31661/gmj.v11i.2562

**Published:** 2022-12-20

**Authors:** Kamran Bagheri Lankarani, Maryam Akbari, Reza Homayounfar, Reza Tabrizi, Mohebat Vali, Mohamad Reza Zakeri, Mojtaba Farjam, Mahmoud Khodadost, Fariba Ahmadizar

**Affiliations:** ^1^ Health Policy Research Center, Institute of Health, Shiraz University of Medical Sciences, Shiraz, Iran; ^2^ National Nutrition and Food Technology Research Institute, Faculty of Nutrition Sciences and Food Technology, Shahid Beheshti University of Medical Sciences, Tehran, Iran; ^3^ Noncommunicable Diseases Research Center, Fasa University of Medical Science, Fasa, Iran; ^4^ Clinical Research Development Unit, Fasa University of Medical Science, Fasa, Iran; ^5^ USERN Office, Fasa University of Medical Sciences, Fasa, Iran; ^6^ Student Research Committee, Shiraz University of Medical Sciences, Shiraz, Iran; ^7^ Department of Public Health, School of Health, Larestan University of Medical Sciences, Larestan, Iran; ^8^ Department of Data Science and Biostatistics, University Medical Center Utrecht, Utrecht, The Netherlands

**Keywords:** Coronavirus 2019, Melatonin, Mortality, C-reactive Protein

## Abstract

The efficacy of melatonin in the treatment of patients with coronavirus 2019 (COVID-19) is controversial. This review has summarized the evidence on the efficacy of oral melatonin compared to placebo in patients with mild to moderate COVID-19 infection. We searched four international online databases and all randomized clinical trials (RCTs) that investigated the effects of melatonin compared with the placebo on clinical outcomes, including mortality, discharge time, O2 saturation (SaO2), and c-reactive protein (CRP) levels in patients with COVID-19 infection, were included. The standard random-effects model with hybrid continuity correction was used to pool the risk ratio (RR), weighted mean difference (WMD), and the I2 index to assess the heterogeneity. A total of 272 patients from five RCTs were included. Our meta-analysis showed melatonin compared to placebo, decreased discharge time (WMD=-0.93 days; 95% confidence interval [CI]:-2.94 to 1.07, P=0.36; I2=56.78%) and the risk of mortality (RR=0.72; 95% CI:0.25 to 2.13, P=0.56; I2=0.0%) in COVID-19 patients. Melatonin intake compared to placebo significantly increased SaO2 (WMD=1.38%; 95% CI:0.09 to 2.68, P=0.04; I2=49.82%) and decreased the CRP levels (WMD=-7.24 mg/l; 95% CI:-11.28 to -3.21, P0.001) in a sensitivity analysis. Our findings showed the efficacy of melatonin compared to placebo in patients with mild to moderate COVID-19 infection.

## Introduction

The most serious complication among patients with coronavirus disease 2019 (COVID-19) is an increased inflammatory response [1]. The clinical characteristics could range from mild symptoms (e.g., diarrhea, headache, cough, shortness of breath, and fever) to more serious conditions, including acute respiratory difficulties, septic shock, and organ failure [2]. Overexpression of the interleukins (IL)-1, IL-6, IL-10, IL-8, tumor necrosis factor-alpha (TNF-), and NOD-like receptor protein 3 (NLRP3) inflammasome causes cytokine storm in patients with acute respiratory distress syndrome and acute lung injury [3][4]. In certain COVID-19 patients, the role of NLRP3 inflammasome activation has been shown in kidney fibrosis and renal and heart failure [5].

There are currently no potential antiviral medications available. Therefore, inhibiting NLRP3 may be particularly important in this condition. Providing low-cost, practical, and readily accessible remedies is critical. Melatonin is a versatile hormone that influences most organ metabolism and affects health and aging [6]. Also, it is the principal neurohormone secreted by the pineal gland and a sleep-wake cycle regulator. Because of its antiapoptotic, immunomodulatory, anti-inflammatory, and antioxidative properties, this chronobiotic medication may be useful against viral infections [7]. Melatonin has historically been used to treat viral infections and respiratory diseases as an immunomodulator and anti-inflammatory treatment [7][8]. Also, melatonin affects various systems, such as normal nervous system aging, neuropathological aging and longevity, circadian rhythm, and mitochondrial metabolism [8]. COVID-19 and other viral pandemics are expected to be best combated by techniques that stimulate and reverse the aging process [9].

Several recent randomized clinical trials (RCTs) have shown that melatonin positively impacts COVID-19 infection [9][10][11][12][13][14][15][16] with inconsistent results. Therefore, we conducted a systematic review and meta-analysis to evaluate the effectiveness of melatonin on the clinical outcomes (mortality and discharge rates, oxygen saturation, and C-reactive protein [CRP] levels) of COVID-19 infection.

## Materials and Methods

This study was conducted based on the Preferred Reporting Items for Systematic Reviews and Meta-Analyzes (PRISMA) guideline [17]. Also, the protocol of this study was registered at PROSPERO (register number:CRD42022306483).

### Search Strategy and Study Selection

A systematic search was performed in four databases, including Cochrane Library, Scopus, PubMed, and Web of Science.

A combination of keywords was used to create the search query in international online databases, e.g. ["melatonin" OR "slenyto," OR "agomelatine,"OR "circadin," OR"rozerem,"] AND ["COVID-19," OR "SARS-CoV-2," OR"coronavirus," OR" 2019-nCoV," OR "corona-virus.] AND ["Mortality" OR "recovery" OR" discharge time" OR" SaO2" OR" SaO2 saturation" OR" CRP" OR " C Reactive protein"] AND ["randomized clinical trials" OR "clinical trial" OR" RCTs"]. Our searches were further restricted to include RCTs investigating the effects of melatonin on primary (includes mortality and discharge time) and secondary (includes O2 saturation [SaO2] and CRP levels) outcomes in patients with COVID-19 disease. The reference list of related RCTs and previous reviews was checked to retrieve any additional studies. All RCTs included were published in the English language up to January 2022. Our study protocol was registered at PROSPERO (register number:CRD42022306483).

### Inclusion and Exclusion Criteria

Studies were selected if they were conducted as original human RCTs (either with cross-over or parallel designs), performed on confirmed COVID-19 infection, investigated the effects of melatonin in the intervention compared to placebo groups, and reported sufficient data on primary and secondary outcomes in both groups. RCTs that did not include a control group and the abstracts of seminars without full papers were excluded from our study.

### Data Extraction

Two independent investigators (MA and RT) extracted relevant data from RCTs using defined forms in Microsoft Excel. Extracted data included the first author's name, year of publication, the basic characteristics of participants, study method, total sample sizes (in intervention/placebo groups), disease, dosage, duration, and type of intervention. The mean (standard deviation [SD]) changes were extracted for discharge time, SaO2 saturation, CRP levels, and the number of mortality in the intervention and placebo groups in each trial. A third author intervened when a disagreement arose (KBL).

### Quality Assessment

The Cochrane Collaboration Risk of Bias Tool [18] was used to assess the methodological quality of included RCTs. Randomization generation, allocation concealment, blinding of participants and result assessors, insufficient outcome data, selective outcome reporting, and other forms of bias were among the criteria used to evaluate this instrument.

### Statistical Analysis

Our studied measures were mean reduction in the time to discharge, CRP level, and improvement in SaO2 in patients with COVID-19. We considered the difference between melatonin and the placebo groups by pooling the weighted mean difference (WMD) using a random-effects analysis in STATA software (Version 12.0; STATA Corporation, College Station, TX, USA).

This meta-analysis used the risk ratio (RR) to compare the mortality rate between the two groups. Due to existing double-zero-event studies in pooling data on mortality, we applied the random-effects model with the hybrid continuity correction in our meta-analysis using "Meta" package in R software Version 6.0-0 (The R Foundation, Boston, MA) [19]. We used the Hartung and Knapp modification to perform a standard random-effects analysis in outcomes with three or fewer three studies for more accuracy. Inter-study heterogeneity was evaluated using Cochran (Q) test and I-square statistic. Significant heterogeneity among studies was considered when I-square exceeded 50% with P<0.1. Several sensitivity analyses were conducted to determine the reliability of the pooled effect sizes after fixing the overall heterogeneity statistical I-square to 25%. The evidence of potential publication bias was statistically assessed using Egger's test in the current meta-analysis.

## Results

**Figure-1:**
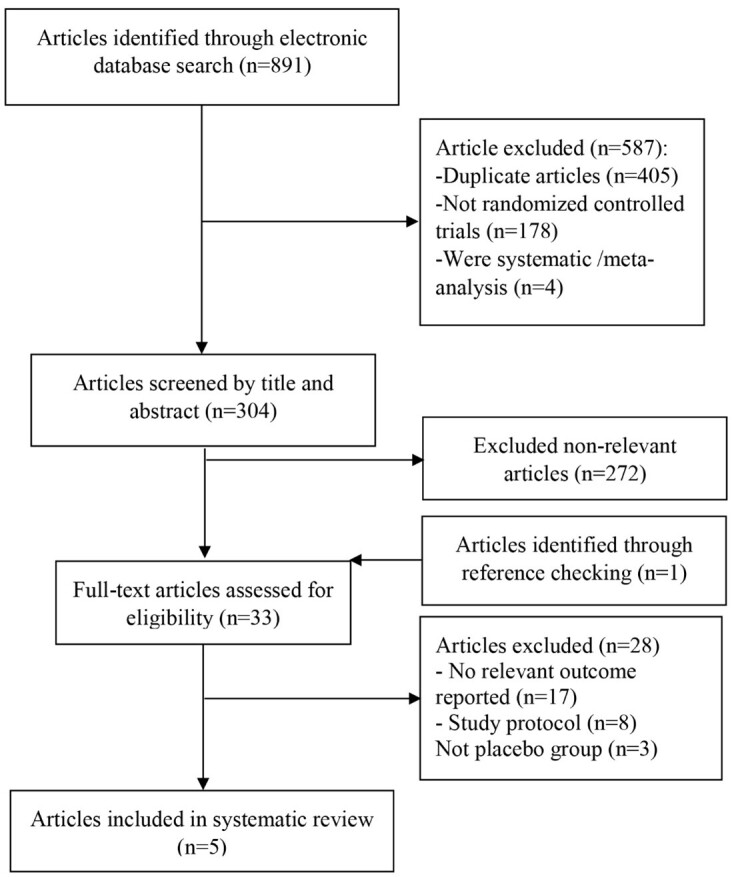
Figure 1. Flowchart of the study identification and selection process

**Table:** Table1. Characteristics of Included Studies

**Authors**	**Sample size (placebo/intervention)**	**Country/patient**	**Duration of intervention**	**Intervention group**	**Study design**	**Outcomes**
**Farnoosh *et al*. [20] **	24/20	Iran/ Mild to moderate	2 week	melatonin (3 mg) three times daily	single-center, randomized, double-blind, placebo, controlled trial	Death, discharge time, CRP
**Alizadeh *et al*. [21] **	14/17	Iran/ Mild to moderate	2 week	melatonin (6 mg) daily	randomized, single-blind, placebo, controlled trial	Death, CRP
**Darban *et al*. [22] **	10/10	Iran/ Sever	10 day	melatonin (6 mg) daily	single-center, randomized, active-controlled, open-label, parallel-group	Death, discharge time, CRP, SaO _2_
**Mousavi *et al*. [23] **	48/48	Iran/ Sever	1 week	melatonin (3 mg) a single nightly	Open‐label, randomized, placebo, controlled trial	Death, discharge time, CRP, SaO _2_
**Davoodian *et al*. [24] **	42/39	Iran/ Mild to moderate	2 week	melatonin (3 mg) three times daily	randomized, double-blind placebo, controlled trial	CRP, SaO _2 _

**CRP:** C Reactive protein

### Characteristics of Studies

**Figure-2:**
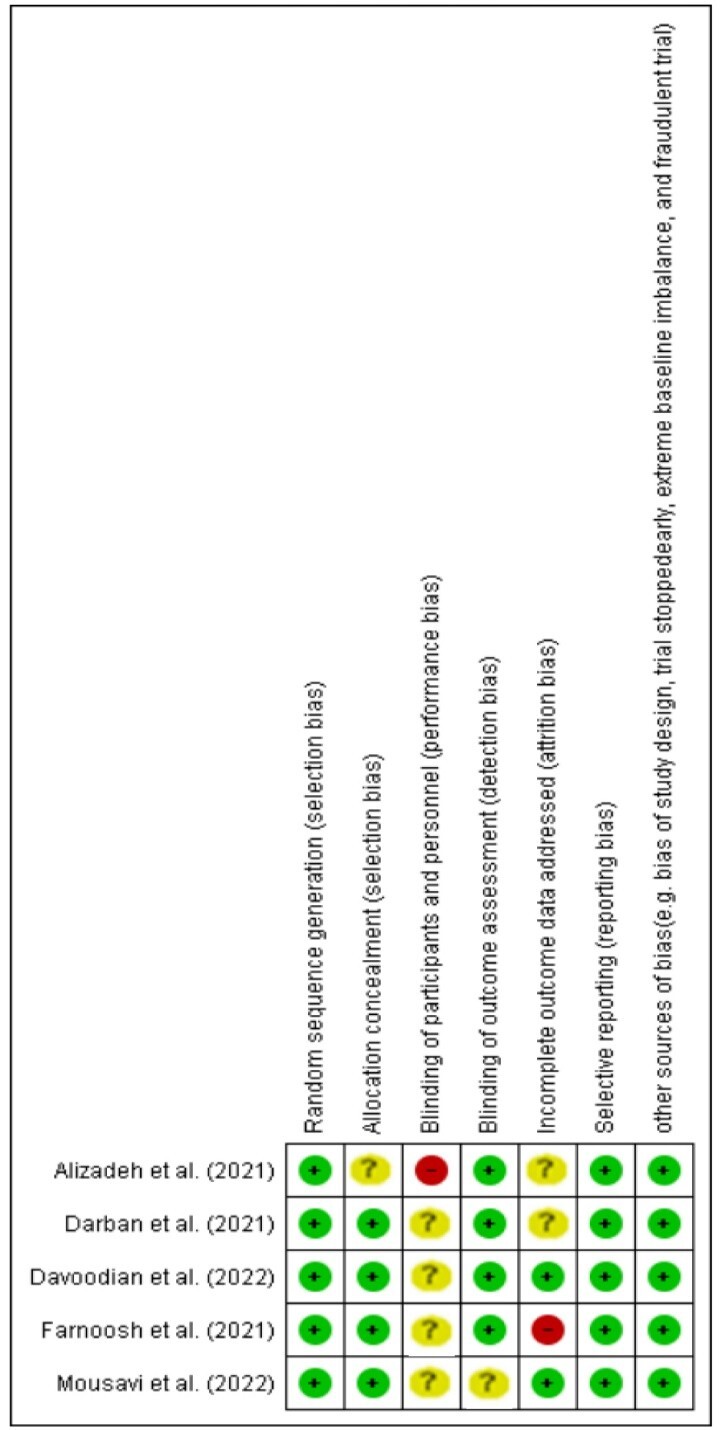
Figure 2. The quality assessment of included studies.

In the current study, a total number of 891 studies was identified through an electronic database search. After excluding the duplicates, systematic reviews, and studies that were not RCTs, 304 studies remained (Figure-1). Also, 272 articles were identified as non-relevant when assessed by title and abstract. After assessing 33 full texts for eligibility, we excluded 17 RCTs with non-relevant outcomes, eight study protocols, and three studies with no placebo group. Finally, we enrolled five RCTs [20][21][22][23][24]; among them, five studies were on CRP [20][21][22][23][24], four on death [20][21][22][23], and three on discharge time and SaO2 as the outcomes that were assessed after melatonin or placebo treatment [22][23][24]. Table-1 depicts the main characteristics of the included trials in the current meta-analysis. The results of the risk of bias for each study are shown in Figure-2.

### Primary Outcomes

**Figure-3:**
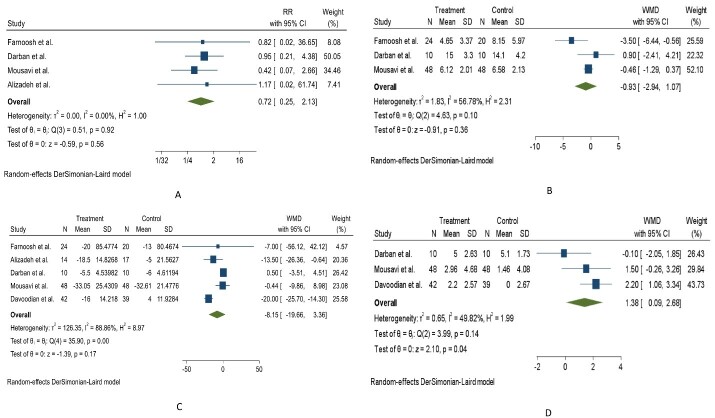
Figure 3. The effects of melatonin on mortality (A), discharge time (B), C reactive protein (CRP) (C), SaO2 (D) in patients with COVID-19

Using a random-effects model, our meta-analysis showed melatonin had a non-significant effect in the reduction of the risk of mortality in COVID-19 patients (RR=0.72; 95% CI:0.25 to 2.13, P=0.56; I2=0.0% [with 4 RCTs]). Moreover, according to the random-effects model, there was a non-significant decrease in discharge time (WMD=-0.93 days; 95% CI:-2.94 to 1.07, P=0.36; I2=56.78% [with 3 RCTs]) in the melatonin group compared with the placebo group (Figure-3A and B).

There was significant heterogeneity among included RCTs (I2=56.78%, Q=4.63, P=0.1). After fixing the overall heterogeneity statistical I2 to 25% in sensitivity analysis, we found no significant change in the reliability of the pooled WMD on discharge time (WMD=-0.78 days; 95% CI:-2.09 to 0.54, P=0.25). Due to the lower number of trials on discharge time, using the Hartung and Knapp modification, our pooled WMD did not significantly change (WMD=-0.93 days; 95% CI:-5.79 to 3.93, P=0.5).

As shown in Table-2, stratifying RCTs based on potential moderator variables, including medical state (severe vs. mild-to-moderate), dosage, and duration of treatment, did not significantly reduce primary outcomes across trials.

### Secondary Outcomes

As shown in Figure-3C, COVID-19 patients randomly assigned to melatonin treatment had a non-significant improvement in CRP levels compared to placebo (WMD=8.15 mg/l; 95% CI:-19.66 to 3.36, P=0.17; I2=88.86% [with 5 RCTs]).

There was significant inter-study heterogeneity between included trials on CRP levels (I2= 88.86%, Q=35.90, P<0.001). After adjusting for statistical heterogeneity to 25%, the sensitivity analysis revealed melatonin significantly reduced CRP levels (WMD=-7.24 mg/l; 95% CI:-11.28 to -3.21, P<0.001). Using the Hartung and Knapp modification for SaO2, we found a significant change in the pooled WMD (1.38%; 95% CI:-1.47 to 4.23, P=0.17).

Subgroup analyses showed that trials with longer duration of treatment (WMD=-18.80 mg/l; 95% CI:-23.98 to -13.62, P<0.001), trials taking a higher dosage of melatonin (WMD=-19.83 mg/l; 95% CI:-25.49 to -14.16, P<0.001), and as well as trials with the mild-to-moderate medical state (WMD=-18.80 mg/l; 95% CI:-23.98 to -13.62, P<0.001) estimated greater benefits of melatonin treatment on decreasing CRP levels compared to placebo (Table-2).

Melatonin intake compared to placebo significantly increased SaO2 (WMD=1.38%; 95% CI:0.09 to 2.68, P=0.04; I2=49.82% [with 3 RCTs], Figure-3D). However, subgroup analyses did not show significant changes in SaO2 across included trials (Table-2).

### Publication Bias

Regression-based Egger’s test indicated no significant evidence of potential small-study effects between included trials for mortality (P=0.91), discharge time (P=0.9), CRP (P=0.97), and SaO2 (P=0.08) in the current meta-analysis.

## Discussion

**Table:** Table2. Subgroup Analysis of the Effects of Melatonin in COVID-19 Patients.

	**Parameters**		**Effect size (95% CI)**	**P-value**	**Heterogeneity (I^2^, P-value) **
	Duration	<2 weeks	0.69 (0.21 to 2.22)	0.528	0.00%, 0.506
		≥2 weeks	0.98 (0.06 to 15.13)	0.986	0.00%, 0.899
**^a^** **Mortality**	Dosage	<9 mg	0.72 (0.23 to 2.21)	0.56	0.00%, 0.776
		≥9 mg	0.82 (0.02 to 36.65)	0.92	-
	Medical state	Mild-to-moderate	0.98 (0.06 to 15.13)	0.986	0.00%, 0.899
		Severe	0.69 (0.21 to 2.22)	0.528	0.00%, 0.506
	Duration	<2 weeks	-0.38 (-1.18 to 0.42)	0.354	0.00%, 0.435
		≥2 weeks	-3.5 (-6.44 to -0.56)	0.02	-
**^b^** **Discharge time**	Dosage	<9 mg	-0.38 (-1.18 to 0.42)	0.354	0.00%, 0.435
		≥9 mg	-3.5 (-6.44 to -0.56)	0.02	-
	Medical state	Mild-to-moderate	-0.38 (-1.18 to 0.42)	0.354	0.00%, 0.435
		Severe	-3.5 (-6.44 to -0.56)	0.02	-
	Duration	<2 weeks	0.36 (-3.33 to 4.05)	0.85	0.00%, 0.857
		≥2 weeks	-18.8 (-23.98 to -13.62)	<0.001	0.00%, 0.593
**^b^** **CRP**	Dosage	<9 mg	-2.51 (-9.36 to 4.34)	0.473	51.83%, 0.125
		≥9 mg	-19.83 (-25.49 to -14.16)	<0.001	0.00%, 0.606
	Medical state	Mild-to-moderate	-18.8 (-23.98 to -13.62)	<0.001	0.00%, 0.593
		Severe	0.36 (-3.34 to 4.05)	0.850	0.00%, 0.857
	Duration	<2 weeks	0.76 (-0.81 to 2.32)	0.342	29.92%, 0.232
		≥2 weeks	2.2 (1.06 to 3.34)	<0.001	-
**^b^** **SaO _2_**	Dosage	<9 mg	0.76 (-0.81 to 2.32)	0.342	29.92%, 0.232
		≥9 mg	2.2 (1.06 to 3.34)	<0.001	-
	Medical state	Mild-to-moderate	2.2 (1.06 to 3.34)	<0.001	-
		Severe	0.76 (-0.81 to 2.32)	0.342	29.92%, 0.232

**CRP:** C Reactive protein; **CI:** Confidence interval ^a^Effect size was considered as risk ratio (RR). ^b^Effect size was considered as weighted mean difference (WMD).

Melatonin had a non-significant effect on decreasing the risk of death and reducing discharge time in COVID-19 patients. Also, melatonin had no significant advantage in lowering CRP levels compared to the placebo. However, our sensitivity analysis found that melatonin significantly lowered CRP levels after controlling for statistical heterogeneity to 25%.

Melatonin intake raised SaO2 substantially when compared to placebos. We discovered a considerable shift in the pooled WMD using the Hartung and Knapp adjustment for SaO2. Subgroup analysis revealed that studies with a longer treatment period, a higher melatonin dose, and mild-to-moderate medical conditions had more robust melatonin therapy advantages than placebo only in lowering CRP levels.

With the increasing prevalence of COVID-19 infection worldwide, knowledge of optimal treatment strategies is essential. Currently, treatment for COVID-19 infection is still experimental, and medications are being administered compassionately. The effectiveness of melatonin as adjunctive therapy in several diseases (such as cancer, influenza, and sepsis) was observed [7][25][26][27].

In addition to continuing medicinal attempts, scientists have been interested in melatonin, a multifunctional chemical, for months, owing to its anti-inflammatory, antioxidant, and immune-modifying properties. Previous studies revealed that melatonin has acceptable positive effects against sleep disturbances, respiratory illnesses, atherosclerosis, and viral infections (such as respiratory syncytial virus, Venezuelan equine encephalitis virus, hepatitis, and Ebola) [8][28][29]. Although the role of melatonin in bat antiviral immunity is unclear, it appears to play a potential role against COVID-19 infection through various routes [8][30].

It was reported that melatonin alleviates oxidative stress induced by viral infections by reduction levels of malondialdehyde 8-isoprostane, boosting antioxidant enzyme activity, and alleviating respiratory symptoms [20][31].

Our study showed that melatonin intake could reduce mortality in the treatment group, but this rate was insignificant. The current study results are similar to the two previous studies [23][32]. Also, studies have shown that melatonin can help people with mild to severe symptoms of COVID-19, such as cough, shortness of breath, and fatigue.

During COVID-19 infection, these are the most common symptoms of lower respiratory tract infections, with a high incidence and death rate. Although there was no significant difference in discharge rates in our study, patients who received melatonin had a faster discharge and returned to baseline.

Based on the available evidence and our findings, melatonin can be a useful supplement for COVID-19 patients. Furthermore, a considerable increase in blood oxygen saturation in the melatonin group compared to the placebo group might be related to the influence of melatonin on oxygen transport and usage in tissues, which has been examined experimentally and clinically [33][34][35][36]. Reduced pulmonary infiltration might also be due to decreased vascular permeability [23].

CRP is a protein marker for inflammation, infection, and tissue damage in the acute phase. With a half-life of 19 hours, IL-6 primarily stimulates hepatic CRP production. High CRP levels are connected with a bad prognosis, and CRP measurement is a reliable diagnostic marker for early screening and fast isolation of patients suspected of developing COVID-19 disease [37][38]. Regarding the current study, CRP levels improved in most patients with significant differences between the two groups, demonstrating that melatonin has a beneficial anti-inflammatory impact on COVID-19 infection.

Overall, the findings imply that using melatonin as a bridge to recovery may lower the COVID-19 disease burden and healthcare use and diminish the efficacy of antiviral medicines as a bridge to recovery, which may have negative effects. Melatonin usage has been linked to fewer adverse effects and dose-limiting toxicity [20][39].

Our study has some limitations. First, the number of RCTs that have examined the effect of melatonin on COVID-19-related outcomes is limited, which can affect the results, especially on oxygen saturation. While the dose of melatonin in studies is 3 and 6 mg, studies with higher doses of melatonin were recommended. High doses of melatonin may show more beneficial effects, especially the effect of melatonin on variables that showed weakness in this study. Although it reduced variables such as death, it did not show significant significance. Also, the follow-up time in RCTs included in our review is short. A longer follow-up may better reveal the effects of melatonin on COVID-19-related outcomes. The high heterogeneity of the studies is also one of the limitations of this study though it has been controlled according to subgroup analyses.

## Conclusion

Our study showed the efficacy of melatonin as adjunctive therapy compared to placebo in controlling COVID-19 disease. Since melatonin is cheap, safe, and easily accessible medicine, it is suggested that future research look into this drug alone or in combination to reduce COVID-19-related consequences.

## Conflict of Interest

The authors declare no competing interests.
